# Lifestyle Behaviors and Depressive Symptoms in Chinese Adolescents Using Regression and fsQCA Models

**DOI:** 10.3389/fpubh.2022.825176

**Published:** 2022-03-22

**Authors:** Songli Mei, Jianping Lv, Hui Ren, Xinmeng Guo, Cuicui Meng, Junsong Fei, Tongshuang Yuan, Jingyi Yue, Ren Gao, Qianqian Song, Xixi Zhao, Yu Ao, Yumei Li

**Affiliations:** ^1^Department of Social Medicine and Health Management, School of Public Health of Jilin University, Changchun, China; ^2^Nursing Department, The First Hospital of Jilin University, Changchun, China; ^3^Department of Pediatric Intensive Care Unit, The First Hospital of Jilin University, Changchun, China

**Keywords:** adolescent, depressive symptom, qualitative comparative analysis models, physical activity, sedentary time, sleep duration, physical education class, screen time

## Abstract

The study was to compare the performance of the regression models and fuzzy set qualitative comparative analysis (fsQCA) models in analyzing the possible effects of sociodemographic variables (age and sex) and lifestyle behaviors (physical activity, sedentary time, sleep duration, physical education class and screen time) on depressive symptoms in adolescents. This cross-sectional surgery was conducted among 726 adolescents in Jilin Province of China, selected by random cluster sampling. The regression model showed that age, physical education (PE) class and sleep duration were associated with depressive symptoms. Meanwhile, the fsQCA models showed that shorter physical activity, PE class and sleep duration as well as longer sedentary and screen time were related to higher levels of depressive symptoms. Both regression and fsQCA models indicated that promoting lifestyle behaviors could affect depressive symptoms. Considering the differences between the two methods, they are not superior to the other method, but complementary and should be used in other studies at the same time.

## Introduction

Depressive symptoms are major and impairing global public health challenge affecting millions worldwide. The first onset of depressive symptoms is often in adolescence (1). The existing body of research has indicated that the prevalence of depressive symptoms among adolescents ranges from 4 to 41% (2). Increasing evidence suggested that depressive symptoms negatively effect on adolescents (3, 4). It not only brings long-term psychological painful experience to adolescents, but also produces a variety of adverse consequences, such as interpersonal problems, substance abuse and suicide (5). In addition, depressive symptoms in adolescence can persist and recur into adulthood, increasing the risk of severe depressive symptoms in adulthood (6). In view of the high prevalence and disease burden of depressive symptoms in adolescents, it is necessary to identify important risk factors and take effective interventions.

There are many factors affecting depressive symptoms, such as living conditions, life events and lifestyle factors (7). Among them, lifestyle factors, such as sleep duration, physical activity, physical education (PE) class participation, screen time and sedentary time could lead to a greater effect on depressive symptoms than previously believed (7–9). A recent meta-analysis showed that although the existing evidence base was still low, universal multi-risk lifestyle interventions may have a modest but statistically significant effect on reducing depressive symptoms (10). These behaviors may jointly affect adolescents' psychological health (11). Recently, people have realized that these lifestyle factors are interdependent and should be considered at the same time (7, 12). For example, the 24-h movement guidelines have been proposed (13). In addition, these factors are often combined in complex ways to affect mental development of adolescents. Therefore, it is crucial to consider the joint occurrence of these factors and their relationship with mental health.

Recently, the combination of lifestyle factors has attracted extensive attention and recognition. Nevertheless, most of the literature focus on linear model (8), which predicts the dependent variable from multiple independent variables, and there is a linear relationship between the predictor variable and predicted variable. It can show the contribution of single variable, but cannot consider the interaction or combination of various variables and the way where different paths may result in the same results. The above problems can be supplemented by fuzzy set qualitative comparative analysis (fsQCA) models (14), which are based on Boolean logic to identify a series of causal conditions associated with a result. Since the fsQCA model is according to the combination of various variables and takes into account equality, that is, different paths leading to the same results (15). They analyze the linear relationships between relevant conditions and results to provide more accurate and comprehensive results (16). These two methods are complementary (17). Therefore, in recent years, increasing studies have gradually proved that the simultaneous use of the two methods helps to understand the relationship between variables more comprehensively and deeply, and fully recognize the possible impact of the combination of these variables on mental health (18–20). Notably, this method is still limited in assessing the relationship between lifestyle behaviors and mental health.

Understanding the relationship between lifestyle behavior and the risk of depressive symptoms is very important to better reduce the prevalence of depressive symptoms and reduce the burden of related diseases. The purpose of the present study was to analyze the effect of sociodemographic variables and lifestyle behaviors on depressive symptoms, comparing two different methodologies: regression and fsQCA.

## Materials and Methods

### Participants

The sample made up of 800 adolescents from 8 junior high schools and 3 senior high schools in Jilin province, China. This study adopted random cluster sampling. Firstly, we randomly selected 8 junior high schools and 3 senior high schools, each school has 3 grades. Secondly, each grade selected about 25 participants and took these students as the research participants. After eliminated the invalid ones, 726 questionnaires were collected with an 90.75% of effective recovery rate. All the questionnaires were answered anonymously and collected on site. The identity and information about participants were strictly protected by researchers. This study was approved by the Ethics Committee of the School of Public Health of Jilin University.

### Measures

#### Depressive Symptom

Depressive symptom was assessed using the Center for Epidemiological Studies Depression Scale (CES-D) complied by the Nation Institute of mental health in 1977 (21). It measures the frequency and severity of depressive symptoms during the past week. It consists of 20 items and four dimensions: somatic activity/inactivity, depressed affect, absence of positive affect and interpersonal difficulties. The scale uses a 4-point Likert scale answer format, ranging from 0 (rarely or none of the time) to 3 (most or all of the time). The total score is 0–60. The higher the total score, the higher the level of depressive symptom. In this sample, the Cronbach's alpha was 0.91.

#### Lifestyle Behaviors

Physical activity was assessed with the question: “In the past 7 days, how many days have you been able to do at least 60 minutes of moderate or high-intensity exercise that increased your heart rate or breathing rate, such as running, playing basketball, playing football, or swimming, and so on?” Sedentary time was assessed by responses to the question, “How many hours do you usually spend sitting in each day?” PE class was assessed by asking: “During this school year, how many PE classes did you have on average every week on campus?” Screen time was measured using the question “How many hours do you spend on using screens a day on average?” Sleep duration was calculated by measuring the hours of sleep habitually at night.

### Statistical Analysis

The software IBM SPSS Statistics 24.0 was used to perform hierarchical regression, and fsQCA 2.5 software (22) was used for fsQCA.

First, the study estimated the descriptive analyses. Second, since calibration was one of the advantages of fsQCA, we also calculated calibration values. Then, regression models and fsQCA were conducted concurrently. For hierarchical regression models (HRM) analysis, two steps were considered for the exploring of depressive symptoms: age, sex (step 1) and five lifestyle behaviors (step 2). Before performing the fsQCA, raw data were converted to fuzzy set and all variables were recalibrated with values between 0 and 1.

fsQCA models determine the percentage of variance explained, or the applicability of the model, coverage, goodness of fit and consistency indicators (16). In fsQCA models, when a condition must exist for a given result, it is considered necessary, and its consistency is ≥0.90 (16). When the consistency is about 0.75 or more, the condition is considered sufficient (17); for a given result, it does not always exist and means a combination of conditions resulting in a specific result. The fsQCA 2.5 software (22) recalibrated the values of age, physical activity, sedentary time, PE class, screen time and sleep duration. fsQCA can calibrate important variations for each condition through external and internal criteria. In detail, we calibrated each causal condition with 10, 50, and 90% of the specified values, corresponding to anchors for full membership, the crossover point, and full non-membership (23).

Once the responses were transformed, necessary and sufficient condition tests were used to assess the impact of age and lifestyle behaviors on depressive symptoms. In order to determine the sufficient conditions, the fsQCA analysis included two steps (17): first, the member scores of fuzzy-set were converted into a truth table, listing all logically possible combinations of causal conditions and the empirical results of each configuration. Secondly, fsQCA analysis produced three possible solutions: complex, parsimonious, and intermediate. The last one (the one presented here) was recommended (16).

## Results

### Descriptive Statistics and Calibration Values

All participants were age 10–19 years, with an average of 13.79 (*SD* = 1.64). In terms of sex distribution, 55.2% were males (401) and 44.8%were females (325). As for the educational level, 78.4% (569) participants were junior high school, while only 21.6% (157) were senior high school. The main descriptors and calibration values for the variables of the study were shown in Table 1. The score of depressive symptoms ranged from 0 to 52 points. The average was 13.22 (*SD* = 9.65).

**Table 1:** Main descriptions and calibration values.

	**Age**	**Depressive symptoms**	**Physical activity**	**Sedentary time**	**PE class**	**Screen time**	**Sleep duration**
*M*	13.79	13.22	3.11	8.39	2.33	2.56	8.03
*SD*	1.64	9.65	2.28	2.10	0.86	2.02	1.01
Min	10	0	0	3.7	0	1	6
Max	19	52	7	12	5	12	10.5
**Calibration values**						
P10	12	3	0	5.33	1	1	6.67
P50	13	12	3	8.50	2	2	8.00
P90	16	26	7	10.69	3	5	9.50

*PE Class, Physical education class; M, mean; SD, standard deviation; Min, minimum; Max, maximum; P10, 10th percentile; P50, 50th percentile; P90, 90th percentile*.

### Hierarchical Regression Models

In Table 2, age and sex significantly increased the variance by 2% (ΔR^2^ = 0.02; *p* < 0.001), including the lifestyle behaviors, accounted for 4% of the variance (ΔR^2^ = 0.04; *p* < 0.001). On the one hand, age (β = 0.09; *p* < 0.05) showed statistically significant and positive beta coefficients for depressive symptoms. On the other hand, PE class (β = −0.08; *p* < 0.05) and sleep duration (β = −0.11; *p* < 0.01) showed a significant negative beta coefficient for depressive symptoms.

**Table 2:** Hierarchical regressions for the depressive symptoms.

**Variable**	**Depressive symptoms**	
**Predictors**	**ΔR^**2**^**	**β**
Step1	0.02^***^	
Age		0.14^***^
Sex		0.07
		
Step2	0.04^***^	
Age		0.09^*^
Sex		0.06
Physical activity		−0.06
Sedentary time		−0.03
PE class		−0.08^*^
Screen time		0.03
Sleep duration		−0.11^**^

* *p ≤ 0.05*;

** *p ≤ 0.01*;

*** *p ≤ 0.001. PE Class, Physical education class*.

### Comparative Qualitative Analysis of Fuzzy Sets (fsQCA)

#### Necessary Analysis

Based on the results from fsQCA (Table 3), there was no necessary condition exceeded the consistency threshold of 0.90 as a necessary condition (16).

**Table 3:** Necessity analysis for depressive symptoms.

	**Depressive symptoms**		**~** **Depressive symptoms**	
	**Cons**	**Cov**	**Cons**	**Cov**
Age	0.57	0.71	0.54	0.58
~Age	0.66	0.63	0.73	0.59
Female	0.43	0.52	0.47	0.48
Male	0.57	0.55	0.53	0.45
Physical activity	0.62	0.63	0.69	0.60
~Physical activity	0.61	0.70	0.58	0.56
Sedentary time	0.56	0.64	0.59	0.58
~ Sedentary time	0.63	0.64	0.63	0.55
PE class	0.44	0.66	0.53	0.68
~PE class	0.79	0.66	0.74	0.53
Screen time	0.67	0.67	0.63	0.54
~Screen time	0.53	0.63	0.61	0.61
Sleep duration	0.59	0.64	0.67	0.62
~Sleep duration	0.65	0.70	0.61	0.56

*Cons, consistency; Cov, coverage; ~, absence of condition; Condition needed, consistency ≥0.90. PE Class, Physical education class*.

#### Sufficiency Analysis

In the sufficient analysis, under the premise of fsQCA, the obtained models provided the following results. When the consistency is about 0.75 or more, the model is informative (17).

For high levels of depressive symptoms (Table 4), 6 pathways were observed, explaining 42% of the cases with high levels of depressive symptoms (Overall Consistency = 0.82; Overall Coverage = 0.42). The most relevant path or combination to explain depressive symptoms was the result of the interaction of low physical activity, high sedentary time, low PE class, high screen time and low sleep duration (Raw coverage = 0.25; explaining 25% of cases with high level of depressive symptoms).

**Table 4:** Summary of the three main sufficient conditions for the intermediate solution of depressive symptoms.

**Frequency cutoff: 1**	**Depressive symptoms**					
	**Consistency cutoff:0.85**					
	**1**	**2**	**3**	**4**	**5**	**6**
Age		•	•		•	•
Female	°	°	°			°
Physical activity	°	°		°	•	°
Sedentary time	°			•	°	•
PE class	°	°	°	°	°	
Screen time			•	•	•	•
Sleep duration	°	°	°	°	°	°
Raw coverage	0.17	0.17	0.18	0.25	0.20	0.11
Unique coverage	0.03	0.01	0.02	0.07	0.04	0.01
Consistency	0.84	0.86	0.84	0.86	0.86	0.89
Overall solution consistency	0.82					
Overall solution coverage	0.42					

•*, presence of condition; °, absence of condition (low levels). PE Class, Physical education class*.

## Discussion

The aim of this study was to explore and compare the influence of sociodemographic characteristics and lifestyle behaviors on depressive symptoms in Chinese adolescents. Moreover, a comparison was conducted to discover strengths and disadvantages of two analytical methods, namely, regression models and fsQCA models.

The regression model suggested that age, PE class and sleep duration were associated with depressive symptoms, as reported in previous studies (24). According to the results of fsQCA analyses, no necessary conditions for depressive symptoms were observed. In terms of the sufficiency analysis, the most important interactions for depressive symptoms were the following conditions: low physical activity, high sedentary time, low PE class, high screen time and low sleep duration.

This result may be because with the increase of age, adolescents feel more academic and social pressure, the changes of interpersonal relationship and physiological factors, which leading to corresponding changes in adolescents' psychology, such as depressive symptom (25).

Adolescent health related risk behaviors in life often do not exist alone, but present gathered or phenomenon. Therefore, fsQCA was used in this study to explore the effect of the combination of different behavior patterns on depression symptoms. Although limited or moderate exposure may help adolescents benefit from devices and screen time, excessive screen time may increase the risk of developing habits that adverse physical and mental health (26). Furthermore, adolescents who use more screen time may also have a tendency to self-isolate (27), which may increase the risk of depressive symptoms. Screen time partially aggravates depressive symptoms by shortening sleep time (28). There was evidence that light-emitting devices may affect sleep quality by dysregulating melatonin secretion (29); in addition, entertainment content on electronic products may stimulate adolescents, thereby prolonging sleep latency and shortening sleep time (30). Adolescents with insufficient sleep are more vulnerable to negative emotions (31). For sedentary time based on screen time, there may be mechanisms for the link between sedentary behavior and depressive symptoms, such as through potential inflammatory pathways and neurotransmitter function (32). These above results also consistent with the self-efficacy theory, that is, physical activity have a significant positive effect on depressive symptoms and anxiety (33). Physical activity can affect depressive symptoms through various physiological, psychosocial and social mechanisms; for example, by activating neural plasticity in brain regions which is associated depressive symptoms, thereby reducing inflammation and regulate emotions (34). Physical activity during PE classes can relieve fatigue and improve pleasure through neurophysiological stimulation and the brain's information processing function (i.e., cerebral cortex), so as to improve children's preparation ability for all day exercise (35, 36). The decrease in the frequency of physical education will affect adolescents' opportunities to develop friendship and social skills, increasing the risk of depressive symptoms (9).

Overall, the observed results seem to support, at least in part, the existing study on the impact of sociodemographic and lifestyle behaviors on depressive symptoms (37). However, most of the existing studies focused on linear relationship models, neglecting the supplement of other methods such as fsQCA models (38). Although these variables are important, if we only focus on regression model, they will be ignored, and they are important related factors when interacting with other conditions. In addition, fsQCA can obtain the same results from different paths. From the perspective of intervention, this is particularly important because there are conditions that we cannot take action, such as age, but others are vulnerable to intervention, such as physical activity.

In short, low physical activity, PE class, sleep duration as well as high sedentary and screen time have been proven harmful for adolescents. That is to say, the development of healthier lifestyle behaviors will lead to the improvement of adolescent depressive symptoms, which also will lead to direct and positive results on mental health during adolescence and adulthood. Our results will help to comprehensively understand the symptoms of depressive symptoms in adolescents and its relationship with lifestyle factors, and help relevant people to formulate lifestyle interventions.

## Strengths and Limitations

In conclusion, this study attempted to explore the relationships between lifestyle behaviors and depressive symptoms in Chinese adolescents. The principal advantage of study was to compare two complementary methods to explore lifestyle behaviors and depressive symptoms of adolescents, which allowed a more comprehensive and reliable evaluation. Notably, fsQCA model provided various pathways to combine related factors in different ways, depending on the relations between variables. Thus, fsQCA methodology could complement traditional regression models.

However, it did not rule out some limitations. One of the limitations was the sample. Since this study was only conducted in Jilin province, it is difficult to generalize the results. Another limitation was that the study used self-reports to collect data. Although anonymity decreased social desirability bias in self-reports, response and recall bias could not be completely controlled. Therefore, it was recommended to use objective external measures in future research.

## Conclusion

Sociodemographic and lifestyle behaviors factors were related to depressive symptoms in adolescents. Based on the regression model used, it was found that age, PE class and sleep duration were related variables of depressive symptoms. In the fsQCA models, other variables such as physical activity, sedentary time and screen time also seem to have important effects. Overall, fsQCA model seems to have better ability than regression models. Meanwhile, it allows us to consider the combination between various variables that may result in the same result while considering a single input. In view of the differences between two methods, they are not superior to the other method, but complementary and should be used in other studies at the same time.

## Data Availability Statement

The original contributions presented in the study are included in the article/supplementary materials, further inquiries can be directed to the corresponding authors.

## Ethics Statement

The studies involving human participants were reviewed and approved by Ethics Committee of Jilin University. Written informed consent to participate in this study was provided by the participants' legal guardian/next of kin.

## Author Contributions

SM: conceptualization and writing-original draft preparation. JL: conceptualization, writing-original draft preparation, and software. HR: writing-original draft preparation and methodology. XG and CM: formal analysis. JF: writing-review and editing. TY: validation. JY and RG: data curation. QS and XZ: methodology. YA: supervision. YL: supervision, writing-review, and editing. All authors contributed to the article and approved the submitted version.

## Funding

This work was supported by Higher Education Research Project of Jilin Province (JGJX2021D14) and Science Research of the Department of Education of Jilin Province (JJKH20220956SK).

## Conflict of Interest

The authors declare that the research was conducted in the absence of any commercial or financial relationships that could be construed as a potential conflict of interest.

## Publisher's Note

All claims expressed in this article are solely those of the authors and do not necessarily represent those of their affiliated organizations, or those of the publisher, the editors and the reviewers. Any product that may be evaluated in this article, or claim that may be made by its manufacturer, is not guaranteed or endorsed by the publisher.
